# Is Struvite a Prebiotic Mineral?

**DOI:** 10.3390/life3020321

**Published:** 2013-04-29

**Authors:** Maheen Gull, Matthew A. Pasek

**Affiliations:** Geology Department, University of South Florida, 4202 E Fowler Ave., SCA 528, Tampa, FL 33620, USA; E-Mail: ambermaheen@yahoo.com

**Keywords:** phosphorus, prebiotic synthesis, struvite, origin of life, phosphate esters

## Abstract

The prebiotic relevance of mineral struvite, MgNH_4_PO_4_·6H_2_O, was studied experimentally as a phosphorylating reagent and, theoretically, to understand the geochemical requirements for its formation. The effectiveness of phosphorylation by the phosphate mineral, monetite, CaHPO_4,_ was also studied to compare to the efficiency of struvite. The experiments focused on the phosphorylation reactions of the minerals with organic compounds, such as nucleosides, glycerol and choline chloride, and heat at 75 °C for about 7–8 days and showed up to 28% phosphorylation of glycerol. In contrast, the compositional requirements for the precipitation of struvite are high ammonium and phosphate concentrations, as well as a little Ca^2+^ dissolved in the water. Combined, these requirements suggest that it is not likely that struvite was present in excess on the early Earth to carry out phosphorylation reactions. The present study focuses on the thermodynamic aspects of struvite formation, complementing the results given by Orgel and Handschuh (1973), which were based on the kinetic effects.

## 1. Introduction

Phosphorus (P) is an important element for life. It is the limiting reagent in many ecosystems and is also ubiquitous in biochemistry, because phosphorylated biomolecules play significant roles in replication and information (as RNA and DNA), metabolism (as ATP, NADPH and other coenzymes) and structure (as phospholipids) [1,2]. The synthesis of organophosphates is a conundrum in prebiotic chemistry, due to the low solubility and reactivity of phosphate minerals in terrestrial environments.

The dominant form of P is as the orthophosphate anion (PO_4_^3−^) and its associated minerals. These minerals, which include the calcium phosphate minerals, apatite, Ca_5_(PO_4_)_3_(F,Cl,OH), brushite, CaHPO_4_·2H_2_O, and whitlockite, Ca_9_MgH(PO_4_)_7_ [3], are poorly soluble and exhibit little reactivity towards organics in water under neutral, low temperature environments [4,5,6,7]. It is also believed that precipitation of brushite might have been possible in the hadean oceans, perhaps due to a higher partial CO_2_ pressure. Another noteworthy mineral for prebiotic phosphorylation consideration is struvite (MgNH_4_PO_4_·6H_2_O). It is believed that certain favorable geological conditions could also precipitate out this mineral [6]; however, it is still unclear if struvite would have been common on the early Earth [8]. It has been shown previously that it is precipitated out when phosphate (PO_4_) is added to seawater containing more than 0.01 M ammonium [9]. This precipitation requires a questionably high concentration of NH_4_^+^ ions [10,11], leading to debate as to its plausibility on the early Earth [9,11]. Indeed, current estimates of ammonia in the early oceans suggest a maximum of about 1 mM [11].

Organic phosphorylation reactions via struvite have been discussed previously [9]; however, not much has been done recently. To address this lack of recent phosphorylation attempts, we investigated the potential prebiotic phosphorylations of glycerol and choline using minerals struvite and monetite (CaHPO_4_) as the source of PO_4_. Monetite was chosen to provide a comparison between phosphorylation using struvite and using a typical calcium phosphate. Previous prebiotic syntheses of organophosphates have used mineral P sources [2,12,13,14], though these utilize different P sources.

## 2. Experimental

### 2.1. Materials

Choline chloride, adenosine, uridine, monetite (CaHPO_4_) and D_2_O were obtained from ACROS, while glycerol was from Alfa Aesar. Monetite was chosen to represent calcium phosphate minerals, as it is the anhydrous form of brushite. Deionized water (DI) was purified in house using a Barnstead (Dubuque, IA, USA) NANO pure^®^ Diamond Analytical combined reverse osmosis-deionization system. Struvite was synthesized by reaction of equal volumes of 0.5 M MgCl_2_, NH_4_Cl and Na_2_HPO_4_ solutions, resulting in a white precipitate that was identified by an Enwave Raman microscope as struvite.

### 2.2. Methods

For studying phosphorylation reactions by mineral struvite, phosphorylation reactions of glycerol were performed by adding 0.3 g struvite into an aqueous solution (about 7 mL of DI water) of 1.5 g glycerol. For choline chloride phosphorylation, again 0.3 g of struvite and 1.39 g of choline chloride were added to 7 mL of DI water. Adenosine and uridine both used 1 g of the organic, with all other materials remaining the same.

For studying phosphorylation reactions by mineral monetite, 0.5 g monetite mineral and 1.5 g glycerol were added into 7 mL of DI water. For choline chloride phosphorylation, 0.5 g of monetite mineral and 1.39 g of choline chloride were added to 7 mL of DI water. Similarly, adenosine and uridine both used 1 g of the organic, with all other materials remaining the same.

The vials were closed and sealed. The reaction vials were heated for 7–8 days at 75 °C. After the completion of the reaction, the samples were filtered or centrifuged and were saved for ^31^P-NMR and MS (mass spectrometric) analysis. 

^31^P-NMR spectra were acquired on a Unity INOVA 400 spectrometer (161.84 MHz for ^31^P and 399.88254 MHz for ^1^H) equipped with a variable temperature controller and a Varian 5 mm Autoswitchable with a z-axis gradient probe optimized for tuning of ^31^P and ^1^H on the first of a series of samples in D_2_O. The ^31^P chemical shifts are reported using an external reference standard (neat solution of phosphoric acid at room temperature (~25 °C, δ ppm = 0.0). A ^31^P 45° flip angle pulse was used for both proton decoupled and non-decoupled spectra (90° ^31^P pulse of 9.8 μs at 54 dB attenuation, where max power output ~300 watts). For proton decoupled spectra, a composite pulse Waltz decoupling sequence was applied with field strength of 2,525 Hz during the acquisition time of 1 s and the relaxation time of 1 s. The signal was an average from 512 transients. All spectra were processed by ACD Labs 12.0 package and or VnmrJ 2.2D or 3.2A. Specific products were identified by determining the H-P coupling constants of individual reaction products, as well as finding specific masses in the mass spectra.

Molecular weights of the target products were taken on LC-MSD Agilent and in negative ESI modes. For P-NMR studies, the samples were simply mixed with D_2_O and analyzed.

### 2.3. Geochemical Modeling

Geochemical computations were carried out using the program HSC (version 7.0, Outokompu Research Oy) (The HSC chemical code stands for enthalpy, entropy and heat capacity. More details on the code can be found at [15]). This code uses the GIBBS energy solver [16] to determine equilibrium concentrations and has been used previously to constrain sulfur chemistry in the solar system [17], the composition of Europa’s ocean [18] and the mineral chemistry under rapid heating conditions [19]. Although the code has the ability to estimate activities by the Davies model (extended Debye-Hückel) theory, this was not done with the present data set for reasons outlined below. The code allows for the injection or removal of species, computing the resulting changes in solution chemistry with respect to time. Reaction rates are not considered by this code, as thermodynamics is time independent. However, kinetics can be mimicked by decreasing the activity of certain species so as to inhibit the formation of these species in a computational solution. 

In this system, the initial abundances of H_2_O were set equal to 10^6^ kg (5.56 × 10^7^ moles). Eighty-five thousand moles of N_2_ gas was added to this system; this number represents the proportionate amount of moles of N_2_ in the atmosphere relative to the total moles of water in the ocean. The salinity was set equivalent to the salinity of seawater for Na, Mg and Ca.

In this system, the dominant anion is Cl^−^. Phosphate concentrations in the water were adjusted by addition of NaH_2_PO_4_ and Na_2_HPO_4_ in a 4:1 ratio (a pH of 7.8). Since the precipitation of struvite and Ca-phosphates is strongly modified by pH, the pH of the ocean water was buffered by adding an excess of H_2_S and Na_2_S, which kept the pH of the solution between 7.6 and 7.9, despite the addition of significant NH_3_. Addition of this buffer precluded accurate activity composition, as the ionic strength of the solution would have been strongly modified by this buffer.

Three things were intentionally changed during this calculation: the amount of phosphate added, the Mg/Ca ratio and the amount of N in NH_3_ or NH_4_^+^. These changes were placed within the model by modifying the NaH_2_PO_4_ and Na_2_HPO_4_ initially present, the MgCl_2_/CaCl_2_ initially present and by increasing the NH_3_ gas while decreasing the N_2_ gas proportionately (going from 0–100% NH_3_). The temperature was kept constant at 298 K and the pressure constant at 1 bar. pH was buffered by the sulfide species listed above. As no other sulfur species were permitted to form beyond H_2_S and HS^−^, no redox or pH chemistry could take place due to oxidation of sulfide to sulfate.

For all the cases considered here, once the abundances of the elements are supplied, the HSC program then calculates the distribution of elements amongst the various chemical species (listed below) by solving the minimum thermodynamic energy state coupled to mass balance calculations. With the code, we tracked the quantities of the following compounds representing phosphates, including CaHPO_3_, CaHPO_4_, Ca(H_2_PO_4_)_2_, CaHPO_4_ × 2H_2_O, Ca(H_2_PO_4_)2 × H_2_O, Ca_2_P_2_O_7_, Ca_3_(PO_4_)_2_, Ca_5_(PO_4_)_3_OH, MgNH_4_PO_4_ × 6H_2_O, Mg_2_P_2_O_7_, Mg_3_(PO_4_)_2_, NaH_2_PO_4_ and Na_2_HPO_4_. Oxides considered in this study included CaO, Ca(OH)_2_, CaMgO_2_, MgO and Mg(OH)_2_. Solutes in water included Ca^+2^, Cl^−^, H_2_, H^+^, HNO_3_, H_3_PO_2_, H_3_PO_3_, H_3_PO_4_, HPO_3_^−2^, HPO_4_^−2^, H_2_PO_2_^−^, H_2_S, HS^−^, H_2_PO_3_^−^, H_2_PO_4_^−^, Mg^+2^, N_2_, NH_3_, NH_4_^+^, O_2_, Na^+^, OH^−^ and PO_4_^−3^. Gases included: N_2_, H, H_2_, H_2_O, NH_3_, HCl and O_2_.

Data for these species come from a variety of different sources and are stored within the HSC database. The data for struvite came from previous reports [20].

The formation of struvite takes place via the reaction:

Mg^2+^ + NH_4_^+^ + PO_4_^3−^ + 6H_2_O = MgNH_4_PO_4_ × 6H_2_O

The precipitation of struvite, as calculated using thermodynamic equilibrium modeling, is dependent on four factors: pH, redox conditions, Mg/Ca ratio and total amount and total phosphate. The pH at, which struvite forms must be conducive to NH_4_^+^ speciation and Mg^2+^ speciation (as opposed to MgOH^+^). For the purposes of this study, the pH was kept between 7.6 and 7.9 by adding a mixture of H_2_S and Na_2_S as buffer compounds.

Ammonia must form, as well, so as to allow for the accumulation of ammonium within water. Ammonia formation is controlled by the fugacity of hydrogen:

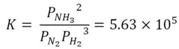


Most of this ammonia, in turn, reacts with water to give aqueous ammonia or ammonium, depending on pH.

Critical to the formation of struvite is a suitable quantity of Mg to react with phosphate and ammonia. However, as calcium phosphates are thermodynamically preferred, the Mg/Ca ratio is critical for estimating the stability range of struvite.

## 3. Results

### 3.1. Experimental Results

**Figure 1:**
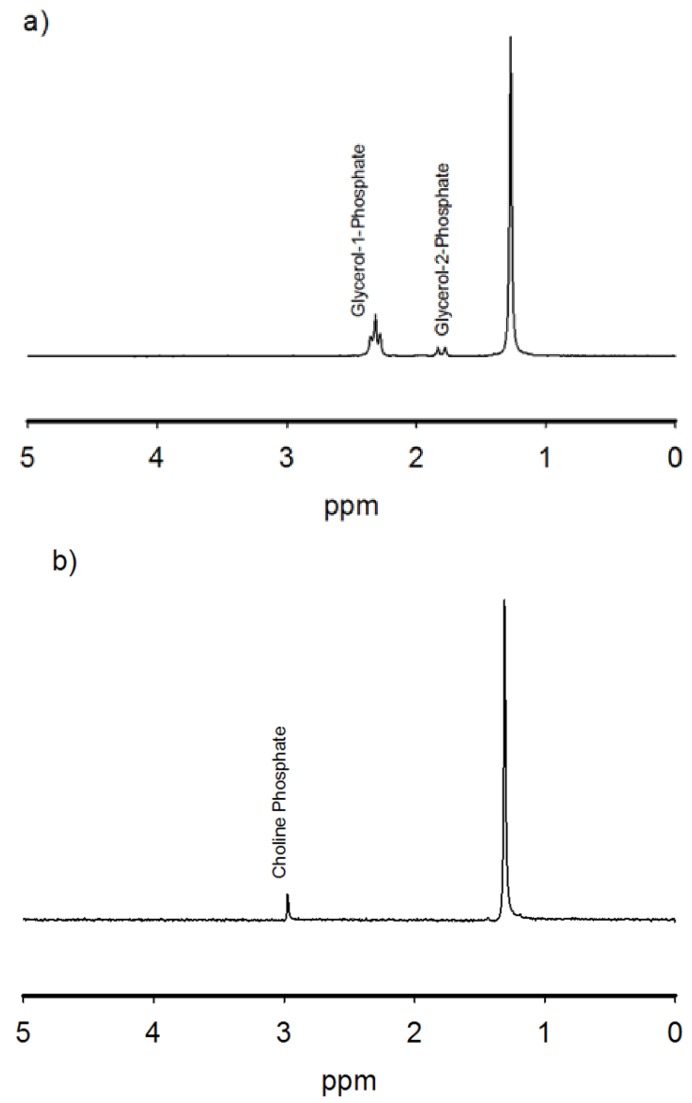
^31^P-NMR spectra demonstrating phosphorylation of (**a**) glycerol and (**b**) choline by struvite. The unlabeled peak (~1.2 ppm) is dissolved orthophosphate.

Products were analyzed by ^31^P NMR (Figure 1), and their molecular masses were confirmed by negative mode ESI-MS. The product phosphocholine was identified by a characteristic peak at *m/z* 183, while glycerol phosphates were identified by the peak at *m/z* 171 (Table 1 and Figure 1). We observed no phosphorylation of adenosine or uridine in any of the experiments.

**Table 1:** Phosphorylation by mineral P, as measured by ^31^P-NMR. CH_2_-O-P linkages are characterized by a triplet when proton coupled, whereas CH-O-P linkages are characterized by doublets. “BDL” means below detection limit.

Reaction	Yield (%) CH_2_-O-P	Yield (%)CH_-_OP
Glycerol + struvite	28 ± 5	6 ± 2
Choline chloride + struvite	5 ± 1	-------
Glycerol + monetite	12 ± 3	BDL
Choline chloride + monetite	BDL	BDL

### 3.2. Geochemical Modeling

The precipitation of struvite takes place primarily over a pH of 6–9 and over this pH region competes directly with the precipitation of calcium phosphate.

**Figure 2:**
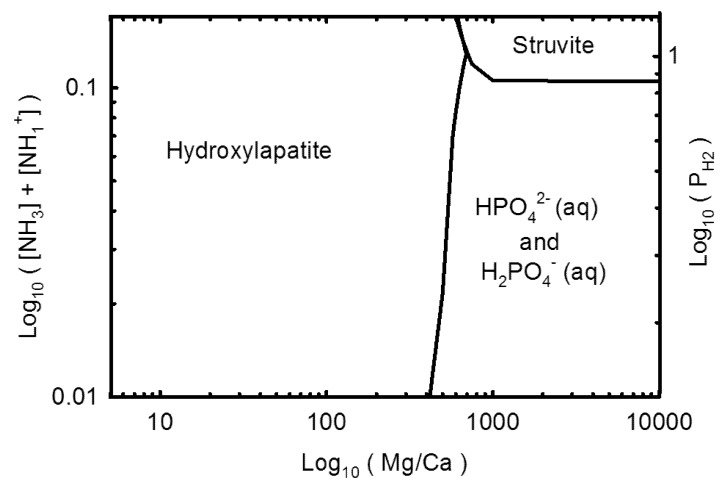
Mg/Ca ratio and ammonia or P_H2_ concentration requirements for struvite precipitation over a pH of 7.6–7.9, at 25 °C, with a phosphate concentration of 10^–5^ M.

Struvite formation is highly dependent on the Mg/Ca molar ratio [21,22]. Based solely on the thermodynamic equilibrium calculations performed here, struvite can never precipitate at the present Mg/Ca ratio, even with the addition of significant ammonia (Figure 2). However, if the Mg/Ca ratio is increased, struvite can be generated at a ratio of about 580 if the phosphate concentration is 10^–5^ M. Under these conditions, struvite and apatite compete for phosphate. At an Mg/Ca ratio of about 700 and greater, struvite is the sole phosphate mineral precipitate. At a large Mg/Ca ratio, struvite begins to form at about 0.1 M ammonium concentration. The redox conditions most conducive to struvite formation are highly reducing (Figure 2) and require a large partial pressure of H_2_.

**Figure 3:**
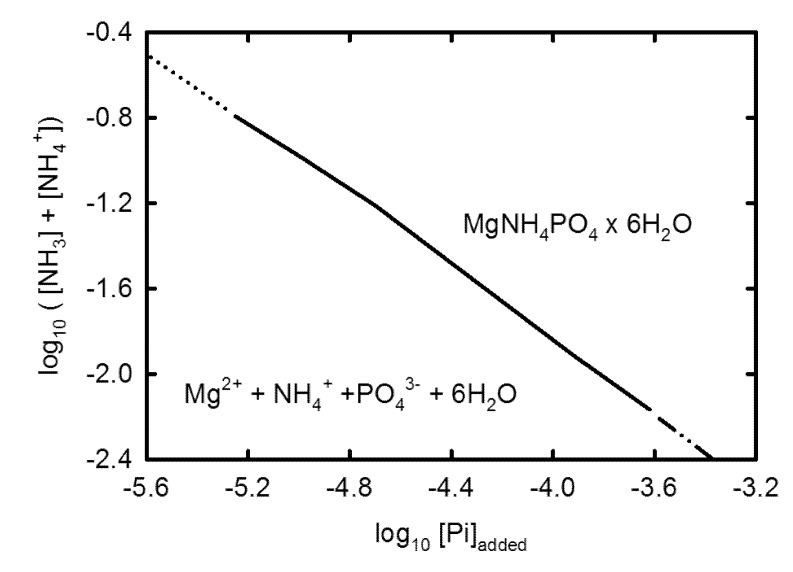
Phosphate and ammonia concentration requirements for struvite precipitation over a pH of 7.6–7.9, at 25 °C, with an Mg/Ca ratio of 10,000. The dotted line represents ammonium concentrations greater than the current quantity of nitrogen in the Earth’s atmosphere, and the dash-dot line occurs where struvite precipitation competes with Mg_3_(PO_4_)_2_ precipitation or farringtonite.

A higher concentration of phosphate can overcome a lower ammonium concentration. If the Mg/Ca ratio is suitably large, then only 0.01 M of ammonium is required for struvite precipitation, with a concentration of phosphate on the order of 10^–4^ M (Figure 3). However, at a very high phosphate concentration, precipitation of struvite competes with precipitation of Mg-phosphates and, hence, struvite requires at least 0.0065 M of ammonium to precipitate at this pH region.

Although temperature was not analyzed in the present study, the solubility of apatite peaks at about 20 °C, whereas the solubility of struvite peaks at about 30 °C [20]. The difference between these two temperatures is not considered to be significant and likely does not alter the P chemistry described here.

## 4. Discussion

These calculations suggest struvite was not present on the early Earth. From the thermodynamic calculations performed here, the formation of struvite requires extremely reducing conditions, a high Mg/Ca ratio and elevated phosphate concentrations.

How then do these results contradict those of Handschuh and Orgel [9], who showed struvite synthesis upon addition of phosphate to ammonia-rich seawater? The calculations performed here ignore kinetic effects. Handschuh and Orgel argued that the system they set up did not reach equilibrium, but instead, Mg interfered with the precipitation of Ca-phosphates, such as apatite. A similar argument had been put forth by Martens and Harriss [23]. This argument is reasonable, as thermodynamic models neglect kinetic effects by nature. However, this suggests that the struvite formed by Handschuh and Orgel may not have persisted for a significant length of time and would slowly convert to calcium phosphates. It is unclear from the experiments of Handschuh and Orgel how long the struvite was in direct contact with the ocean water simulant. The rate of conversion of struvite to apatite is not known, but the sole example from the geological literature of minerals found in penguin rookeries show complete loss of struvite after relatively short amounts of time [24].

Furthermore, these calculations highlight a significant problem in the precipitation experiments of Handschuh and Orgel. The interference of Mg with calcium phosphate precipitation is modeled by decreasing the activity of Ca, causing a high Mg/Ca ratio. However, the concentration of ammonium used by these authors was 0.01 M, to which Na_3_PO_4_ was slowly added. Handschuh and Orgel did not state the total amount of Na_3_PO_4_ added to the solution, only that less than 20% of Ca, Mg and NH_4_ precipitated as a result of phosphate addition. Our calculations show that, at an ammonia concentration of 0.01 M, at least 1.2 × 10^−4^ M of phosphate had to be added. This elevated phosphate concentration is not consistent with typical environmental oceanic conditions, which usually have a factor of 10^−6^ M of phosphate or less [25,26]. Additionally, the high ammonium requirement is questionable given its low predicted abundance in the Hadean ocean [11].

The experimental results show that both struvite and monetite can produce low to mild concentrations of organophosphate, though struvite is a better phosphorylating mineral than monetite. Only glycerol and choline chloride were phosphorylated, which may be expected, as the thermodynamics of phosphorylation of glycerol (and presumably choline) is significantly more favorable than that of nucleosides [2], with a lower energy requirement needed for phosphorylation of glycerol. These results suggest, perhaps, that some of the earliest prebiotic molecules to be phosphorylated may have been membrane-forming molecules, as these molecules are the only ones phosphorylated by simple heating conditions. However, the solubility and availability of phosphate on the early Earth, as shown by the presented calculations, shows that it is unlikely that struvite has been abundant as an efficient P source [8]. Furthermore, even though organophosphates are formed, only a few organic compounds are phosphorylated by these minerals [1,7,17,27].

## 5. Conclusions

Struvite can phosphorylate glycerol and choline to appreciable yields. However, struvite cannot be considered as a potential prebiotic phosphorylating reagent, as it likely was not present on the early Earth. In the modern day geochemical environment, struvite is an exceedingly rare mineral. Major environmental sources of this mineral are all connected to biological production and include bird and bat guano [28], mammalian kidney stones [29] and wastewater effluent [30]. Although the modern Earth is significantly more oxidizing than the ancient Earth, it may be possible to dismiss struvite as a constituent on the early Earth, due to its requirements of an extremely reducing environment, low activity of calcium in water and requirement for elevated phosphate concentrations to yield significant struvite**.** These requirements result from the thermodynamic properties of struvite formation and contrast to Handschuh and Orgel’s (1973) study [9] that focused primarily on the kinetic production of struvite. Additionally, the poor reactivity of both struvite and monetite towards other organic compounds further stresses searching for an alternate efficient source of P for the origin of biological phosphates on the early Earth [1,7,16,31]. 
